# Supplementary material for: Early Nutritional Intervention to Promote Healthy Eating Habits in Pediatric Oncology: A Feasibility Study
Source: Nutrients. 2022 Feb 28;14(5):1024. doi: 10.3390/nu14051024 (PMC8912879; doi:10.3390/nu14051024)
Supplement: Supplementary file 1 [file nutrients-14-01024-s001.zip › nutrients-1577719-supplementary-done.pdf]

nutrients

MDPI

Supplementary Table S1. Comparison of attendance and completion rates of study measures at visits among participants retained in the intervention and those excluded

	Retained	Excluded	
	n = 45	*n* = 16	*p*-Value
Initial evaluation	**Attendance**		
Actual/potential, *n*	45/45	16/16	
Rate (%)	45 (100.0)	(100.0)	-
Study measures	**Completion rates, *n* (%)**		
3-day food record	18 (40.0)	5 (31.3)	0.57
Blood sample	42 (93.3)	13 (81.3)	0.18
24H-R	40 (88.9)	13 (81.3)	0.42
BMI or W/L	45 (100)	16 (100)	-
WC	15 (33.3)	5 (31.3)	1.00
MUAC	29 (64.4)	8 (50.0)	0.38
TSFT	23 (51.1)	6 (37.5)	0.40
SSFT	18 (40.0)	5 (31.3)	0.57
2-month follow-up	**Attendance**		
Actual/potential, *n*	38/45	9/11	
Rate (%)	(84.4)	(81.8)	1.00
Study measures	**Completion rates, *n* (%)**		
24H-R	34 (89.5)	8 (88.9)	1.00
BMI or W/L	38 (100)	7 (77.8)	0.03
WC	7 (18.4)	0	0.82
MUAC	14 (36.8)	5 (50.0)	0.49
TSFT	13 (34.2)	5 (50.0)	0.47
SSFT	11 (28.9)	3 (30.0)	1.00
4-month follow-up	**Attendance**		
Actual/potential, *n*	33/45	5/10	
Rate (%)	(73.3)	(50.0)	0.26
Study measures	**Completion rates, *n* (%)**		
24H-R	29 (87.9)	2 (40.0)	0.04
BMI or W/L	31 (93.9)	4 (80.0)	0.35
WC	3 (9.1)	0	1.00
MUAC	13 (39.4)	1 (20.0)	0.63
TSFT	10 (30.3)	1 (20.0)	1.00
SSFT	7 (21.2)	1 (20.0)	1.00
6-month follow-up	**Attendance**		
Actual/potential, *n*	32/45	3/6	
Rate (%)	(71.1)	(50.0)	0.36
Study measures	**Completion rates, *n* (%)**		
24H-R	25 (78.1)	3 (100)	1.00
BMI or W/L	31 (96.9)	3 (100)	1.00
WC	3 (9.4)	0	1.00
MUAC	8 (25.0)	0	1.00
TSFT	6 (18.8)	0	1.00
SSFT	4 (12.5)	0	1.00
8-month follow-up	**Attendance**		
Actual/potential, *n*	31/45	2/1	
Rate (%)	(68.9)	(50.0)	0.54

Study measures	**Completion rates, *n* (%)**		
24H-R	26 (83.9)	1 (100)	1.00
BMI or W/L	29 (93.5)	1 (100)	1.00
WC	3 (9.7)	0	1.00
MUAC	7 (22.6)	0	1.00
TSFT	7 (22.6)	0	1.00
SSFT	6 (19.4)	0	1.00
10-month follow-up	**Attendance**		
Actual/potential, *n*	22/45	1/1	
Rate (%)	(48.9)	(100)	1.00
Study measures	**Completion rates, *n* (%)**		
24H-R	20 (90.9)	0	0.13
BMI or W/L	21 (95.5)	1 (100)	1.00
WC	1 (4.5)	0	1.00
MUAC	5 (22.7)	0	1.00
TSFT	4 (18.2)	0	1.00
SSFT	1 (4.5)	0	1.00
12-month follow-up	**Attendance**		
Actual/potential, *n*	22/45		
Rate (%)	(48.9)	N/A	-
Study measures	**Completion rates, *n* (%)**		
24H-R	16 (72.7)	N/A	-
BMI or W/L	19 (86.4)	N/A	-
WC	7 (31.8)	N/A	-
MUAC	9 (40.9)	N/A	-
TSFT	7 (31.8)	N/A	-
SSFT	5 (22.7)	N/A	-

Attendance was defined as the number of participants who attended a visit divided by the total number of potential participants. Completion rates of study measures (24H-R, BMI, WC, MUAC, TSFT and SSFT) were calculated based on participants who attended each visit and expressed as percentage. The relationships between feasibility parameters (attendance and study measures completion rates) and retention in the intervention were assessed using Pearson's Chi-Square or Fisher's exact tests. 24H-R: 24-hour recall; BMI: body-mass-index; W/L: weight-for-length ratio; WC: waist-circumference; MUAC: mid-upper arm circumference; TSFT: triceps skinfold thickness; SSFT: subscapular skinfold thickness.
